# Consumer Perception of the Performance of Online Catering Food Safety Regulations: The Case of Shanghai, China

**DOI:** 10.3390/foods13162568

**Published:** 2024-08-17

**Authors:** Weijun Liu, Yige Wu, Yue Sun, Wojciech J. Florkowski

**Affiliations:** 1 College of Economics and Management, Shanghai Ocean University, 999 Huchenghuan Road, Shanghai 201306, China; wjliu@shou.edu.cn (W.L.);; 2 Shanghai Social Survey Center, Shanghai Ocean University Branch, 999 Huchenghuan Road, Shanghai 201306, China; 3 Department of Agricultural & Applied Economics, University of Georgia, 1109 Experiment Street, 212 Stuckey, Griffin, GA 30223-1797, USA

**Keywords:** online meal, food safety regulation, performance, consumer perception, influence factor

## Abstract

To protect the safety of food bought from the online catering sector, the former State Food and Drug Administration of China issued two separate regulations in 2016 and 2017. Independently, the Shanghai government formulated local regulations, including the Shanghai Online Catering Service Supervision and Management Measures, to strengthen food safety supervision in this megacity with the largest catering sector in China. This study explored factors that influence consumer perceptions of the performance of online catering food safety regulations using survey data from 1050 respondents collected in 2019. The results indicate that consumers believe that Shanghai’s online catering industry has improved by varying degrees in terms of food freshness, ingredient traceability, foreign matter control, food temperature control, internal packaging hygiene and environmental protection, and satisfaction with food safety. The factors that influenced the listed features include the number and effectiveness of government-issued regulations regarding the online catering sector, effectiveness of ordering platform food safety regulations, employee training frequency, employee food safety awareness, delivery box cleanliness and courier personal hygiene, consumer trust in ordering platform services, and consumer confidence in government supervision. These factors significantly and positively affected the consumers’ perceptions of the performance of food safety regulations in the online catering sector.

## 1. Introduction

Rapid social and economic development has significantly improved the living standards of the Chinese people, which has led to an increased emphasis on life quality and health. At the same time, rapid technical improvements in the communications industry and the fast development of the Internet have led to the continuous expansion of the online food-ordering industry. However, the explosive growth of the online food service industry has posed severe challenges to food safety. In response to these challenges, the China Food and Drug Administration issued the “Measures for the Administration of Illegal Acts in Online Food Safety” and the “Measures for the Supervision and Administration of Food Safety in Online Catering Services” in 2016 and 2017, respectively, in an attempt to reduce the risks to food safety. Furthermore, the government of the leading urban megacity in China, Shanghai, has also strengthened the supervision of food safety in online catering by issuing local regulations, such as the “Regulations on the Supervision and Administration of Online Catering Services in Shanghai” and the “Shanghai Food Safety Regulations”. The regulations aimed at promoting the standardization of online catering services to ensure public food safety and guide the robust development of online food ordering.

Customers are all too often victims of food safety incidents [1], and thus, they have become an important force behind food safety supervision, while they are also the ultimate beneficiaries of food safety regulations. Their timely feedback on compromised food safety promptly curbs the spread of risks [2]. Therefore, improving the understanding of the role of consumers in food safety monitoring involves studying the consumer perception of the regulatory system and the formulation of science-based regulations to enhance the efficiency and credibility of food safety protection [3]. Furthermore, research indicated [4,5] that the performance evaluation of relevant laws and regulations can effectively improve government efficiency. Examples of performance evaluation include studies in other sectors, such as financial forestry fund expenditures [6], eco-friendly construction [7,8], and performance audit evaluation indicators for local government water management [9].

In recent years, Shanghai consumers’ knowledge and awareness of food safety has substantially improved. Consumers play an important positive role in the system of food safety supervision of the online catering sector [10]. To assess the performance of recently implemented food safety regulations for online catering in China, this study recognized the key role of the customer perspective and investigated Shanghai consumer perceptions of the online catering food safety regulations and performance. Using the survey data collected specifically for this study, an ordered logit regression was applied to empirically examine the effects of the recently changed regulations as perceived by consumers. The ultimate purpose of this study was to generate new knowledge to improve food safety in the online catering sector in Shanghai and to offer suggestions that promote the sustained development of online catering in China through the informed refinement of regulatory measures.

## 2. Literature Review

Several studies focused on consumer and food safety regulations, consumer awareness of food safety [11,12,13], food safety influence on purchase behavior [14,15,16,17,18], and consumer willingness to pay for food safety [19,20,21]. This study explored ways in which key factors affect the consumer perception of the implementation of food safety regulations in the online catering sector pathways and factors.

### 2.1. The Key Food Safety Issues Relevant to the Online Catering Sector

Past studies considered food safety from multiple perspectives. Specifically, Ji analyzed the quality condition, freshness, hygiene, and other factors as important indicators of food safety satisfaction [22]. Yu et al. (2017) identified the freshness of the food and support for the local food system as key factors that consumers considered when purchasing fresh food [23]. Kim and Jung also concluded that food freshness is an essential attribute that influences a purchase [24]. Consumers obtain information about food risks and benefits from the Internet [25], and the traceability system is considered fundamental to the prevention of food safety risks and a food safety information source for consumers [26,27,28,29]. Wang et al. (2009), Wu et al., and Cui et al. found that consumer willingness to pay for food is influenced by the level of trust in traceability labels [20,30,31]. A factor of concern to consumers that influences their consumption behavior is the cleanliness and hygiene of a restaurant [32]. Henson also found that food preparation and cleanliness in restaurants are among the main concerns for customers when dining, and customers pay attention to issues such as whether the food is properly cooked and the presence of foreign matter in the food [33]. Namkung et al. and Wang et al. indicated that the temperature of takeaway food has a significant impact on the food safety and customer satisfaction [34,35,36], which was confirmed by Liu and Lee [37]. Ritchie et al. confirmed that improving the food packaging enhances consumer satisfaction [38]. Additionally, Shuang et al., Hong et al., and Yu et al. found that the hygiene and environmental friendliness of takeaway food packaging materials also affect consumer perceptions and could lead to food safety issues that threaten their health and safety [38,39,40,41,42]. Evidence shows that in the case of a food safety incident, governments at all levels are able to respond calmly in China, actively address issues, and handle problems rationally, which leads to an improvement in the overall safety of the food and operations [43]. Ultimately, this is an expected outcome of increasing emphasis on food safety regulation, where the government and relevant departments continuously enhance the regulatory awareness and enforcement capabilities in the food sector. The food hygiene and safety supervision system has also gradually improved, which has led to an enhanced sensitivity to food safety issues and strengthened market control capabilities.

### 2.2. Food Safety Regulation Studies: Implications for the Online Catering Sector

Research on the performance of food safety regulations in online catering has focused on establishing a comprehensive food safety system [44,45], effective food supervision and management [46,47], and food safety governance [48,49,50]. Several developed countries have established a robust regulatory food safety system for online caterers [44,51,52], while the regulations in developing countries have been slower to emerge [53,54,55]. In China, the majority of research on food safety issues focused on the regulation of traditional catering services, with limited studies on the regulation of online catering services [56,57]. Research on the effects of national or local regulations is also limited, but empirical studies of the policy effects in other areas related to food safety provide valuable insights. Wu et al. [58] examined the effects of government policies on the farm disposal of pig carcasses, where they demonstrated that the effects of various policies differ and interact. Guo et al. [59] researched China’s food safety regulatory system and how it compared with that of other developed countries and concluded that the most important external driver for improving food safety in China is the formulation of new government food safety regulations and the enhancement of old ones. Laar et al. [60] suggested the necessity of improving the domestic conditions to ensure a healthy food supply and establish relevant legislation and leadership.

### 2.3. Factors Influencing the Performance of Food Safety Regulations in Online Catering

Previous empirical studies provide guidance on the factors that influence the performance of food safety regulations in the online catering sector. In addition to considering personal characteristics, such as the consumer’s gender [61], age [62], occupation [63], education [64], and income [65,66,67,68], consumer satisfaction with food safety is an essential indicator [69]. Several studies showed that overall food quality significantly impacts customer satisfaction and behavioral intentions [35,70,71]. Considering the government perspective, Wang et al. [72] and Schoderer et al. [73] suggested that standardization in the food safety regulatory system and ensuring adaptive adjustments are important in its practical implementation. Similarly, the ordering platforms are key players responsible for food safety in online catering. Through standards set in accordance with national regulations, they effectively control the behaviors of relevant parties and reduce food safety risks. They protect consumer rights and enhance consumer satisfaction. From the perspective of merchants, research shows that strengthening food safety training and certification reduces non-compliance behavior [11,74,75,76,77,78]. Additionally, transforming the knowledge of food safety and accountability into awareness reduces the occurrence of foodborne illnesses [79,80,81]. Therefore, training is crucial in food safety practice, and the training of food operators is widely used and was proven effective in enhancing food safety [82,83]. As far as the delivery personnel are considered, Zhang et al. [84] and Li et al. [85] indicate that while the ordering platforms impose performance assessments on delivery couriers, more detailed standards need to be implemented regarding personal hygiene and the hygiene of delivery boxes. Furthermore, consumer trust is essential in food service and crucial in promoting the wide acceptance of food [86,87]. Consumers need to access reliable and trustworthy information to make informed decisions about food and its safety [25]. Bai et al. [88] and Cho et al. [89] found that perceived value and customer satisfaction with food delivery platforms and purchase intentions are greatly influenced by the level of trust, authenticity, and reliability of the products. Consumer satisfaction with food safety and trust in regulations are important indicators of the effectiveness of food safety assurance policy systems and serve as a crucial gauge for government food safety regulations [90]. Zhang and Liu [91] and Yang et al. [92] showed that regardless of consumer satisfaction with food safety, consumers trust the government most of all, and regulations are viewed as being the responsibility of the government and an important guarantee for continued consumer confidence [22].

### 2.4. Summary

The existing literature provides important references for this study, but there are several shortcomings in previous research. First, research on the progress of the implementation of food safety regulations in online catering is lacking. Studies considering how consumers perceive the implementation of food safety regulations in online catering are absent. The rigorous examination of food safety regulations in the online catering sector are not a primary focus in the literature. Previous studies addressed enterprise performance [93,94], with limited attention given to regulatory performance, while exploring environmental performance [95,96], technology policy [97], and auditing [98]. Second, there is a lack of specific micro-level survey data for research. The use of survey data permits the analysis of the dynamic characteristics of variables and enhances the accuracy of estimation results using econometric models.

To fill the existing research gap, this study addressed three aspects. First, using Shanghai as a case study, this paper presents an in-depth study of the efficacy of online catering food safety regulations as perceived by consumers. This study refined the variables involved and focused on the dynamic differences between different stakeholders under the influence of regulations to reflect the multifaceted influencing factors on regulatory performance, thus filling the gaps in the previous research. Second, this study utilized micro-level survey data to describe consumers’ perceptions following the implementation of food safety regulations in online ordering. Third, this study employed an ordered logit regression to analyze the performance of food safety regulations in online catering, demonstrating the effectiveness of regulatory implementation from the consumer’s perspective. Additionally, this paper presents the analysis results of the current status of regulatory implementation and related issues and provides targeted suggestions that may serve as a reference for the government to further improve the regulatory system for food safety in online catering services. They may help to build a more favorable industry ecosystem and make an important contribution to practical research on this subject.

## 3. Data and Methodology

### 3.1. Data

This study collected data through an online survey of consumers in Shanghai. Prior to the survey, consultations with experts from the Shanghai Food Safety Working Committee and the Shanghai Food Society assisted in the preparation of the survey implementation plan and finalizing the questionnaire in early August 2019. The questionnaire included multiple-choice questions, open-ended questions, and questions that included Likert scales, with a focus on the consumers’ awareness of food safety regulations in online catering and their perception of food safety level. The questionnaire was released online in October 2019, with each potential respondent receiving an offer of corresponding points or a gift to incentivize participation. The survey lasted two weeks from 15 October 2019 to 26 October 2019, during which 1162 questionnaires were collected. After eliminating 112 incomplete questionnaires, the sample included 1050 observations, which resulted in a response rate of 90.36%.

Table 1 shows the shares of respondents with regard to selected attributes. As many as 63.05% of respondents were female, 63.05% had Shanghai household registration, 65.43% were between 23 and 40 years of age, and 79.05% had at least a bachelor’s degree. Additionally, the occupations of 49.91% respondents were business management or technical research and development, and 64% had a monthly income of at least RMB 6001. The survey sample was similar to earlier consumer surveys [20] and consisted predominantly of middle-aged, well-educated females with relatively high monthly incomes.

### 3.2. Estimation Approach and Empirical Specification

The issue investigated in this study was the consumer’s perception of food safety regulations in the online catering sector. The choice offered to the survey subjects was a discrete multi-value. Therefore, this study applied the ordered logit and specified the empirical model as follows:

Ln(pk) = θk + βi1gender + βi2houseregist + βi3age + βi4occupation + βi5edulevel + βi6perprice + βi7govnumb + βi8govrationa + βi9platnumb + βi10platrationa + βi11freqtrain + βi12emploaware + βi13boxclean + βi14riderclean + βi15plattrust + βi16govtrust + μi(1)

In Equation (1), pk is the probability of consumer perception of food safety regulations in the online catering sector. θk is the intercept when y = k, where k = 1, 2, 3. The empirical model included 16 control variables: gender (X1), household registration or houseregist (X2), age (X3), occupation (X4), educational attainment level or edulevel (X5), and monthly income or perprice (X6), govnumb (X7) and govrationa (X8) represent the perceptions of the quantity and rationality of government-issued online catering regulations, platnumb (X9) and platrationa (X10) represent the perceptions of the quantity and rationality of regulations set by online ordering platforms, freqtrain (X11) and emploaware (X12) account for the perceptions of restaurant employee training and changes in their food safety awareness, boxclean (X13) and riderclean (X14) capture the perceptions of the hygiene of delivery boxes and the personal hygiene of delivery riders, and plattrust (X15) and govtrust (X16) represent changes in consumer trust in platform services and confidence in the government regulations. Lastly, μi is the estimation error.

### 3.3. Variable Selection

This study specified six dependent variables to reflect the performance of the food safety regulations from the perspective of the consumer. The variables referred to six options presented to a respondent in the questions “Compared to 2017 (2 years ago), what are your perceptions of the approximate changes in ‘food hygiene and safety’ in online food ordering?” and “Compared to 2017 (2 years ago), what are your perceptions of the approximate changes in ‘food safety regulations, supervision, and effects’ in online food ordering?”. The options included “Level of Freshness of Ingredients”, “Level of Ingredient Traceability”, “Level of Foreign Object Control”, “Level of Food Warmth Maintenance”, “Hygiene and Environmental Protection of Inner Packaging”, “Food Safety Satisfaction”, and “Effectiveness of Food Safety Supervision”, and these were directly used as indicators. The response options were set along a five-point Likert-like scale as “Significantly Improved”, “Slightly Improved”, “Remained Basically Unchanged”, “Slightly Declined”, and “Significantly Declined”. After the data review, the responses were grouped into three levels: “Remained Basically Unchanged or Declined”, “Slightly Improved”, and “Significantly Improved”.

The explanatory variables were specified using the responses to several sets of questions. The questionnaire was designed to capture consumer views and features related to the following aspects: “Main Characteristics of Consumers”, “Changes in Regulations-Government Supervision Behavior”, “Changes in Regulations-Platform Behavior”, “Changes in Regulations-Merchant Behavior”, “Changes in Regulations-Delivery Courier Behavior”, and “Changes in Consumer Trust”.

### 3.4. Sample Description

Table 2 shows the definitions and descriptive statistics of the variables selected for the empirical analysis. It provides the specific descriptions and assigned values for the six dependent variables that reflect the performance of food safety regulations in online catering, as well as the 16 independent variables that captured changes in regulatory behavior from the perspectives of sample basic characteristics, government, platforms, merchants, delivery riders, and consumers.

Table 3 shows that the consumer perceptions of the performance of food safety regulations in online catering (dependent variables) had means between 1.5 and 2. This suggests that from the consumer perspective, there was room for improvement. Specifically, the highest perception mean score, at 1.928, was associated with the hygiene and protection of inner packaging (Y5). The next highest means were associated with the food temperature maintenance (Y4), at 1.867, and satisfaction with food safety (Y6), with a mean of 1.78. However, the consumers perceived the regulations as less effective regarding the freshness of ingredients (Y1), foreign matter control (Y3), and ingredient traceability (Y2), where the corresponding means were 1.659, 1.642, and 1.57, respectively.

## 4. Ordered Logit Estimation Results and Marginal Effects

### 4.1. Ordered Logit Results

The results in Table 3 show that the perceived factors, including the government’s issuance of the number of food safety regulations for online catering (X7), reasonableness of food safety regulations issued by the government (X8), frequency of staff training by restaurant owners (X11), restaurant staff food safety awareness (X12), cleanliness of courier delivery boxes (X13), courier personal hygiene (X14), trust in platform services (X15), and confidence in government supervision (X16), often positively influenced each of the six dependent variables.

The order logit estimation results reported in Table 3 show the statistical significance of variables included in the six empirical relationships that modeled the consumer perceptions of regulations that pertained to food safety in the online ordering sector. Overall, the personal characteristics played a modest role in influencing the consumers’ perceptions. Female respondents perceived that the temperature control of ordered foods was not affected by regulations. The three consumer attributes significantly influenced the perception of the effect of regulation on ingredient traceability. Having Shanghai registration status and being older was associated with perceiving that the regulations improved the traceability of ingredients, but those with more education felt the regulations did not enhance the traceability.

The number and effectiveness of government food safety regulations increased the perception of the effectiveness of recent regulations in all empirical models except in the case of the level of control of foreign matter presence in the food (Table 3). The effectiveness of the platform regulations was significant in the case of ingredient traceability and satisfaction with food safety equations. However, the variable that reflected the trust consumers had in the platform services positively influenced their perceptions that the recent regulations improved the ingredient freshness, traceability, foreign matter presence in the food, temperature maintenance, and the level of satisfaction with the food safety overall. Having confidence in government supervision increased the perceptions of effectiveness, which had positive effects in the cases of ingredient freshness, traceability, and two features of couriers that delivered the ordered food (Table 3). Furthermore, the consumer views of courier personal hygiene improvement increased the perception of regulations that enhanced all six factors of the online ordering sector considered in this study, and five aspects (except the ingredient traceability) were enhanced by the view of improved courier delivery box cleanliness. The staff training frequency was positively associated with the perception of regulations that improved the freshness of the ingredients, control of foreign matter, and the maintenance of proper food temperature (Table 3).

The estimated coefficients lacked direct practical interpretation and were converted into probabilities (Table 4). The calculated changes discussed in the next section show the probabilities of the level of perceptions regarding online food safety regulations in response to changes in the explanatory variables.

### 4.2. Marginal Effects

The review of marginal effects (Table 4) showed the general pattern of the probability of consumer perceptions of the regulatory changes as “slightly improving” or “significantly improving” food safety in the online ordering sector. The pattern also revealed that the probability of perceiving the regulations as having had negative effects or lacked an effect on food safety perceptions decreased in response to changes in the explanatory variables. There were only two exceptions to the described pattern, which involved the educational attainment effect on the ingredient freshness variable and the effect of being a female on the ordered food temperature maintenance variable (Table 4). The discussion of the results of each modeled relationship provides details on all the statistically significant probability changes.

#### 4.2.1. Ingredient Freshness

The probability changes that measured the effect of explanatory variables associated with the perception of regulations “slightly improving” food safety in online catering sector tended to be substantially larger than those that indicated “significant improvement”. The probability of consumers that perceived “slight improvement” increased by 3.4% for the consumers that reported a Shanghai registration status. The probability increase associated with the same category (“slight improvement”) were of similar magnitude for the effectiveness of government food safety regulations (3.6%), employee training frequency (3.3%), courier deliver box cleanliness (3.5%), courier personal hygiene (3.1%), and trust in ordering platform services (3.2%). The noticeably largest probability change in that response category was associated with the number of government-issued food safety regulations (9.2%).

The probabilities of consumers that perceived that the regulatory changes “significantly improved” food safety ranged from the lowest at 1.7% in the case of courier personal hygiene to 2.0% in the case of the effectiveness of government food safety regulations to the highest probability change of 5.2%, which was associated with the number of government-issued regulations (Table 4). The probability changes that decreased the consumer perception of negative or indifferent (no change) effects of recently implemented regulations of food safety in the online ordering sector tended to exceed the positive effects. These probabilities ranged from the −14.4% associated with the number of government food safety regulations to the −4.8% associated with the influence of courier personal hygiene.

#### 4.2.2. Ingredient Traceability

The pattern of probability changes regarding the consumer perceptions of recent food safety regulations indicates that the probability of viewing the regulations as having “slightly improved” food safety in online catering sector tended to be substantially higher than the probabilities of seeing the regulations as having “significantly improved” ingredient traceability (Table 4). Specifically, the largest probability change was associated with having Shanghai registration status, at 3.9%, and the lowest with the courier personal hygiene, at 2.7%. The probabilities of consumers that perceived that the regulations “significantly improved” the ingredient traceability ranged from the 3.1% associated with Shanghai registration to the 2.1% linked to courier personal hygiene. However, the exception was the effect of consumer education, which showed the opposite change in the probability, namely, consumers with a higher educational attainment had a lower probability of viewing the recent changes in food safety regulations and the probability of agreeing that the regulations had “slightly” or “significantly” improved ingredient traceability decreased by 2.5% and 2.0%.

The effect of education was consistent in the case where the regulations did not improve or negatively affect the ingredient traceability, which increased by 4.4% (Table 4). This result was important because education influenced the consumer behavior and food consumption. It appeared that there was a need to further research the effectiveness of regulations on ingredient traceability and reasons for the public’s perception of their effectiveness because this was the only statistically significant result of this nature in all six equations. The probability that the new regulations did not improve the ingredient traceability decreased such consumer perceptions by about 6.9% in the cases of consumers that had Shanghai registration status and a restaurant employee food safety awareness level. The probabilities decreased by 4.8% to 5.6% in response to a change in the number of government-issued food safety regulations, effectiveness of ordering platform regulations, trust in platform services, and courier personal hygiene (Table 4).

#### 4.2.3. Foreign Matter Control

The control of foreign matter in food is necessary for consumer safety, and several variables increased the probability of consumer perceptions that the recent regulatory changes enhanced the safety of food sold by the online catering sector. The employee training frequency and employee food safety awareness increased the probability that consumers perceived the recent regulations as having “slightly improved” the safety of online ordered food by 2.6% and 4.3%, respectively (Table 4). The corresponding increases in the probabilities of consumer perceptions of “significant improvement” were 2.0% and 3.3%, respectively, in response to these two variables. The cleanliness of the courier delivery box and the courier’s personal hygiene improvement also increased the probability of consumers viewing the recent regulatory changes as having enhanced food safety. The probabilities increased the perceptions linked to “slight improvement” and “significant improvement” by 3.9% and 3.0%, respectively (Table 4). Trust in ordering platform services also increased these probabilities by 2.7% and 2.2%, respectively. In a pattern similar to the results of the earlier-discussed relationships, the probabilities associated with the opinion that regulations did not improve or left food safety at the same level were negative and lowered the chances of such perceptions by 4.6% to 7.6% for the statistically significant variables (Table 4).

#### 4.2.4. Food Temperature Maintenance

The results show that six explanatory variables significantly influenced the consumer perceptions classified in three levels of food safety improvement with regard to online catering regulations (Table 4). In contrast to the results from the three earlier models, the probabilities associated with the “significant improvement” were of a larger magnitude than those linked to a “slight improvement” and all of them increased the consumer perceptions of the better temperature control of ordered food. The largest probability increases that involved “significant improvement” were linked to the effectiveness of government regulations, at 6.1%, and courier personal hygiene, at 4.7%. The number of government-issued regulations increased such a probability by 4.2%, while the restaurant employee training frequency increased this probability by 3.6%. The probabilities of increasing the perceptions that there had been “slight improvement” in the food temperature maintenance were in the range of 1–1.5%, with the somewhat larger probability associated with the effectiveness of government-issued regulations at 2.0% (Table 4). The probability decreases associated with consumers not seeing an improvement in food temperature maintenance ranged from 4.1% with respect to the trust in ordering platform services to 8.0% in the case of the influence of government regulation effectiveness.

#### 4.2.5. Hygiene and Inner Packing Material

Among the variables that increased the probability of consumers perceiving the positive influence of regulations on the hygiene and inner packing material were, not surprisingly, those that measured the restaurant employee food safety awareness and the two associated with the hygiene of the courier delivery box and the courier’s personal hygiene. Employees that came in direct contact with the ordered meal as it was prepared for delivery and their awareness of food safety increased the probability of the consumer perception of regulations that enhanced the hygiene and inner packing material, namely, a 5.3% “slight improvement” and 3.4% “significant improvement” (Table 4). Regarding the two features associated with the courier, the probability increases were particularly higher in the case of “significant improvement” of hygiene and inner packing material linked to food safety: a 8.3% probability increase in the case of delivery box cleanliness and 4.6% probability increase in the case of courier personal hygiene.

The number and effectiveness of government regulations caused the probability of perceiving a “slight improvement” in hygiene and inner packing material to increase by only 1.0% and 0.5%, but increased the significant improvement probabilities by 6.4% and 3.2%, respectively (Table 4). Confidence in government supervision caused the probability of “slight improvement” to increase by 8.8% and by 5.5% in the case of “significant improvement”. The statistically significant variables caused the probability decrease of the perception that the new regulations did not change or worsened the food safety associated with the hygiene and inner packing material. The probability decreases ranged from 3.7% in the case of the effectiveness of government food safety regulations to 9.6% in the case of courier delivery box cleanliness.

#### 4.2.6. Satisfaction with Food Safety

Six explanatory variables increased the probability of “slight improvement” and “significant improvement” associated with satisfaction with food safety in the online catering sector (Table 4). The increased number of government regulations increased the satisfaction with food safety “slightly” by 3.5% and “significantly” by 2.9%, while decreasing the perception of declined or unchanged satisfaction by 6.4% (Table 4). The restaurant employee awareness of food safety increased the probability of satisfaction with food safety “slightly” by a relatively small percent, at 2.1%, and even less, at 1.7%, in the case of “significant improvement”. The delivery box cleanliness and courier personal hygiene increased the consumer satisfaction with food safety by 5.2% and 4.6%, respectively, in the cases of “slight improvement”, and by 4.3% and 3.7% in the case of “significant improvement”. Trust in the ordering platform services increased the probabilities of “slight improvement” in satisfaction with food safety by 6.8% and “significantly improvement” probability by 4.9% (Table 4). Finally, having confidence in government supervision increased the probabilities of “slight improvement” in satisfaction with food safety by 4.3% and “significant improvement” by 3.5%.

## 5. Conclusions and Recommendations

This study attempted to fill the gap in research on the consumer perceptions of regulations aimed at the enhancement of food safety purchased from the online catering sector in Shanghai, China. This study examined the influences of factors and their probabilities of changing consumer perceptions with regard to six areas related to the safety of food prepared and delivered by the online catering sector.

The survey data permitted the estimation of six relationships using the ordered logit regression. The estimated coefficients were converted into probabilities that measured the influence of statistically significant explanatory variables on perceptions that the regulations improved food safety in the online catering sector with respect to ingredient freshness, ingredient traceability, foreign matter control, ordered food temperature maintenance, hygiene of inner packing material, and overall satisfaction with the ordered food.

The socio-demographic characteristics of the surveyed consumers infrequently influenced the consumer perceptions of regulations. Older respondents felt the traceability of ingredients improved, while female respondents noticed the improved food temperature control. However, the most important result was the declined perceptions of regulatory improvement in the case of traceability in response to the increased level of respondent education.

The results indicate that the number and effectiveness of government online catering regulations increased the perception of improvement in all areas except for foreign matter control in food and ingredient traceability and overall consumer satisfaction, respectively. The reasonableness of regulations formulated by online platforms, frequency of training for restaurant staff and their food safety awareness, cleanliness of delivery boxes and personal hygiene status of delivery riders, trust in platform services, and confidence in government supervision all had significant positive effects on the performance of food safety regulations in online catering as perceived by the consumers. The other factors did not show significant effects.

The frequency of restaurant staff training increased the perceptions that three important aspects of food safety improved following the regulations and included the ingredient freshness, foreign matter control, and maintenance of food temperature. The training of staff could reduce the amount of time spent preparing and handling food, which previously caused delays in filling the incoming orders; therefore, emphasizing on-the-job training methods could ensure the efficiency of operations. Teaching restaurant managers on-the-job training methods opens a possibility for appropriate government agencies to assist with efforts to ensure food safety in the online catering sector. Employee food safety awareness is a related factor that was confirmed to increase the consumer perceptions of regulations that improved ingredient traceability, foreign matter control, and inner packing material hygiene, among others. A rising awareness of food safety is reflected in how employees source and handle ingredients. Public education programs can remind the employees and consumers of the risks of compromising food safety. The content of programs and ways of implementing them can be shared by the local and national governments.

The cleanliness of delivery boxes and the courier’s personal hygiene had major influences on the perception of improvement of several of the six aspects of food safety in this study. The majority of restaurants used self-employed couriers and had limited control over the behavior of those couriers or the equipment they use. Efforts to standardize courier appearance and requirements seem to have markedly improved in view of the result. Although not revealed by the survey data, innovative training methods and formal supervision are usually necessary for interventions to have lasting effects.

National and local governments should periodically review the existing regulations because of the rapid growth in the online catering sector. The use of traditional retail outlets is declining, and consumers are increasingly relying on the order delivery format. Timely updates in response to emerging threats to food safety in the online catering sector will sustain the consumers’ trust in the authorities’ ability to prevent food contamination and incidents of illness. Efforts should be made to develop the capacity of regulatory systems that guide food manufacturing and distribution to more adequately accommodate the increasing scale of the online marketplace.

However, even the best regulatory system does not fully guarantee food safety, which suggests that consumers also should continue to educate themselves about food safety and actively engage with the improvement of online catering. They could contribute to the regulatory processes of online food safety by communicating safety-related issues to platform managers and authorities to make them aware of potential risks.

## Figures and Tables

**Table 1:** Description of sample characteristics (N = 1050).

Sample Characteristics	Sample Size	Percentage (%)
Male	388	36.95
Female	662	63.05
Registered in Shanghai	662	63.05
Not registered in Shanghai	388	36.95
23–40 years old	687	65.43
Other ages	363	34.57
Junior college or less	220	20.95
Bachelor’s degree	683	65.05
Master’s degree or higher	147	14.0
Business management or technical research and development position	524	49.91
Other position	526	50.09
Monthly income		
Less than RMB 2000	130	12.38
RMB 2001–4000	90	8.57
RMB 4001–6000	158	15.05
RMB 6001–8000	192	18.29
RMB 8001–12,000 or more	480	45.71

**Table 2:** Selected descriptive statistics of data.

Name	Definition and Units	Mean	Std. Dev.	Min	Max
Dependent Variables					
Ingredient freshness (Y1)	Fresh appearance of ingredients ^a^	1.659	0.631	1	3
Ingredient traceability (Y2)	Records of food source ^a^	1.57	0.693	1	3
Foreign matter control (Y3)	Level of control for food debris and foreign objects ^a^	1.642	0.677	1	3
Food temperature maintenance (Y4)	Food temperature ^a^	1.867	0.741	1	3
Hygiene and environmental protection level of inner packaging (Y5)	Hygienic inner packaging and protection from elements ^a^	1.928	0.763	1	3
Food safety satisfaction (Y6)	Overall satisfaction with food safety in online catering ^a^	1.78	0.618	1	3
Explanatory Variables					
Gender (X1)	Gender: 1 = male, 0 = female	0.37	0.483	0	1
Household registration (X2)	1 = Shanghai, 0 = other area	0.63	0.483	0	1
Age (X3)	1 = 23–40 years old, 0 = other age	0.654	0.476	0	1
Occupation (X4)	1 = business management or technical research and development; 0 = other	0.499	0.5	0	1
Education (X5)	1 = junior college and below; 2 = bachelor’s degree; 3 = master’s degree or higher	1.93	0.587	1	3
Monthly income (X6)	1 = RMB 2000 or less; 2 = RMB 2001–4000; 3 = RMB 4001–6000; 4 = RMB 6001–8000; 5 = RMB 8001–12,000 or more	3.764	1.418	1	5
Number of government-issued food safety regulations for online catering (X7)	Number of food safety regulations and measures issued by the government for online food ordering ^a^	1.93	0.661	1	3
Effectiveness of government-issued food safety regulations for online catering (X8)	Effectiveness of food safety regulations and measures issued by the government for online food ordering ^a^	1.964	0.696	1	3
Number of platform-issued food safety regulations for online catering (X9)	Number of food safety management measures for online food ordering issued by the delivery platform ^a^	1.745	0.717	1	3
Effectiveness of platform-issued food safety regulations for online catering (X10)	Effectiveness of food safety management measures for online food ordering issued by the delivery platform ^a^	1.698	0.669	1	3
Frequency of restaurant staff training on food safety by restaurant owners (X11)	Frequency of organizing food safety training for restaurant staff ^a^	1.536	0.659	1	3
Restaurant staff food safety awareness (X12)	Food safety awareness of restaurant staff ^a^	1.682	0.7	1	3
Cleanliness of courier delivery box (X13)	Cleanliness and hygiene of delivery boxes used by couriers ^a^	1.61	0.697	1	3
Courier personal hygiene (X14)	Personal hygiene and cleanliness of clothing of delivery couriers ^a^	1.703	0.726	1	3
Trust in platform services (X15)	Trust in food safety services provided by the delivery platform ^a^	1.864	0.73	1	3
Confidence in government supervision (X16)	Confidence in government supervision of food safety in online catering sector ^a^	1.63	0.483	1	2

^a^ Scale 1 = remained basically unchanged or declined, 2 = slightly improved, 3 = significantly improved.

**Table 3:** Ordered logit regression results of consumer perceptions of food safety regulations in online catering sector.

Ologit	(1)	(2)	(3)	(4)	(5)	(6)
	Freshness Level of Ingredients (Y1)	Traceability Level of Ingredients (Y2)	Foreign Object Control Level (Y3)	Food Warmth Maintenance Level (Y4)	Hygiene and Environmental Protection Level of Inner Packaging (Y5)	Food Safety Satisfaction (Y6)
Gender (X1)	−0.028 (0.136)	0.009 (0.133)	−0.190 (0.134)	−0.348 *** (0.127)	−0.152 (0.127)	0.114 (0.142)
Household registration (X2)	0.272 ** (0.137)	0.322 ** (0.135)	0.205 (0.135)	0.040 (0.127)	0.118 (0.128)	0.132 (0.143)
Age (X3)	0.151 (0.158)	0.291 * (0.155)	0.132 (0.154)	0.097 (0.146)	0.161 (0.147)	−0.097 (0.164)
Occupation (X4)	0.185 (0.151)	0.116 (0.146)	0.097 (0.148)	−0.110 (0.139)	−0.014 (0.140)	−0.100 (0.157)
Education (X5)	−0.154 (0.115)	−0.207 * (0.112)	−0.042 (0.113)	0.069 (0.107)	0.070 (0.107)	0.099 (0.120)
Monthly income (X6)	−0.010 (0.060)	−0.025 (0.058)	−0.056 (0.058)	0.063 (0.056)	0.008 (0.055)	0.016 (0.062)
Number of government-issued food safety regulations (X7)	0.745 *** (0.127)	0.249 ** (0.121)	0.185 (0.123)	0.275 ** (0.117)	0.392 *** (0.116)	0.403 *** (0.130)
Effectiveness of government-issued food safety regulations (X8)	0.293 ** (0.118)	−0.082 (0.115)	0.267 ** (0.116)	0.401 *** (0.109)	0.198 * (0.111)	0.041 (0.123)
Number of platform-issued food safety regulations for online catering (X9)	0.029 (0.113)	0.171 (0.109)	0.169 (0.111)	0.022 (0.105)	0.021 (0.104)	0.172 (0.119)
Effectiveness of platform-issued food safety regulations (X10)	0.039 (0.119)	0.243 ** (0.117)	0.192 (0.118)	0.087 (0.111)	−0.038 (0.115)	0.221 * (0.127)
Frequency of restaurant staff training on food safety by restaurant owners (X11)	0.266 ** (0.120)	0.063 (0.116)	0.229 ** (0.115)	0.237 ** (0.112)	−0.128 (0.112)	−0.068 (0.126)
Restaurant staff food safety awareness (X12)	0.055 (0.114)	0.317 *** (0.109)	0.380 *** (0.110)	−0.049 (0.106)	0.205 * (0.106)	0.235 ** (0.120)
Cleanliness of courier delivery box (X13)	0.285 ** (0.122)	0.073 (0.122)	0.344 *** (0.120)	0.229 ** (0.113)	0.507 *** (0.114)	0.604 *** (0.130)
Courier personal hygiene (X14)	0.247 ** (0.114)	0.223 ** (0.113)	0.226 ** (0.113)	0.311 *** (0.107)	0.279 *** (0.108)	0.524 *** (0.121)
Trust in platform services (X15)	0.254 ** (0.112)	0.259 ** (0.109)	0.243 ** (0.110)	0.206 ** (0.104)	0.135 (0.105)	0.683 *** (0.120)
Confidence in government supervision (X16)	0.267 ** (0.110)	0.203 * (0.109)	0.128 (0.109)	0.079 (0.104)	0.338 *** (0.103)	0.492 *** (0.117)
cut1	4.081 ***	3.146 ***	3.819 ***	2.729 ***	2.841 ***	4.974 ***
	(0.397)	(0.377)	(0.388)	(0.356)	(0.357)	(0.424)
cut2	7.343 ***	5.222 ***	6.455 ***	4.928 ***	4.949 ***	9.061 ***
	(0.453)	(0.402)	(0.428)	(0.381)	(0.381)	(0.511)
N	1050	1050	1050	1050	1050	1050
Brant test *p* > chi2	0.457	0.442	0.056	0.895	0.269	0.125

Notes: t statistics in parentheses. * *p* < 0.10, ** *p* < 0.05, *** *p* < 0.01.

**Table 4:** Marginal effects results of consumer perceptions of food safety regulations in online catering.

**Variable Name**	**Ingredient Freshness (Y1)**			**Ingredient Traceability (Y2)**			**Foreign Matter Control (Y3)**		
	**(1)**	**(2)**	**(3)**	**(1)**	**(2)**	**(3)**	**(1)**	**(2)**	**(3)**
Gender (X1)	0.00534	−0.00342	−0.00192	−0.00191	0.00106	0.000845	0.0382	−0.0214	−0.0168
Household registration (X2)	−0.0527 **	0.0337 **	0.0190 **	−0.0691 **	0.0385 **	0.0306 **	−0.0412	0.0231	0.0181
Age (X3)	−0.0293	0.0187	0.0105	−0.0625 *	0.0348 *	0.0277 *	−0.0265	0.0148	0.0116
Occupation (X4)	−0.0358	0.0229	0.0129	−0.0249	0.0138	0.0110	−0.0195	0.0110	0.00858
Education (X5)	0.0298	−0.0191	−0.0107	0.0444 *	−0.0247 *	−0.0197 *	0.00850	−0.00477	−0.00374
Monthly income (X6)	0.00190	−0.00122	−0.000685	0.00543	−0.00302	−0.00241	0.0113	−0.00632	−0.00496
Number of government-issued food safety regulations (X7)	−0.144 ***	0.0923 ***	0.0519 ***	−0.0534 **	0.0297 **	0.0237 **	−0.0373	0.0209	0.0164
Effectiveness of government-issued food safety regulations (X8)	−0.0567 **	0.0363 **	0.0204 **	0.0177	−0.00986	−0.00785	−0.0536 **	0.0300 **	0.0235 **
Number of platform-issued food safety regulations for online catering (X9)	−0.00556	0.00356	0.00200	−0.0368	0.0205	0.0163	−0.0339	0.0190	0.0149
Effectiveness of platform-issued food safety regulations (X10)	−0.00751	0.00481	0.00270	−0.0523 **	0.0291 **	0.0232 **	−0.0386	0.0216	0.0169
Frequency of restaurant staff training on food safety by restaurant owners (X11)	−0.0515 **	0.0330 **	0.0185 **	−0.0136	0.00755	0.00601	−0.0460 **	0.0258 **	0.0202 **
Employee food safety awareness (X12)	−0.0107	0.00685	0.00385	−0.0682 ***	0.0380 ***	0.0302 ***	−0.0763 ***	0.0428 ***	0.0335 ***
Cleanliness of courier delivery box (X13)	−0.0553 **	0.0354 **	0.0199 **	−0.0157	0.00874	0.00696	−0.0692 ***	0.0388 ***	0.0304 ***
Courier personal hygiene (X14)	−0.0479 **	0.0306 **	0.0172 **	−0.0479 **	0.0267 **	0.0212 *	−0.0455 **	0.0255 **	0.0200 **
Trust in platform services (X15)	−0.0493 **	0.0315 **	0.0177 **	−0.0557 **	0.0310 **	0.0247 **	−0.0489 **	0.0274 **	0.0215 **
Confidence in government supervision (X16)	−0.0518 **	0.0331 **	0.0186 **	−0.0436 *	0.0243 *	0.0193 *	−0.0258	0.0145	0.0113
**Variable Name**	**Food Warmth Maintenance Level (Y4)**			**Hygiene and Environmental Protection Level of Inner Packaging (Y5)**			**Food Safety Satisfaction (Y6)**		
	**(1)**	**(2)**	**(3)**	**(1)**	**(2)**	**(3)**	**(1)**	**(2)**	**(3)**
Gender (X1)	0.0691 ***	−0.0165 **	−0.0526 ***	0.0288	−0.00396	−0.0248	−0.0180	0.00993	0.00810
Household registration (X2)	−0.00794	0.00190	0.00605	−0.0224	0.00308	0.0193	−0.0210	0.0116	0.00945
Age (X3)	−0.0193	0.00461	0.0147	−0.0305	0.00420	0.0263	0.0154	−0.00849	−0.00692
Occupation (X4)	0.0219	−0.00522	−0.0167	0.00273	−0.000376	−0.00236	0.0158	−0.00872	−0.00711
Education (X5)	−0.0138	0.00329	0.0105	−0.0133	0.00182	0.0114	−0.0157	0.00865	0.00705
Monthly income (X6)	−0.0126	0.00301	0.00959	−0.00150	0.000206	0.00129	−0.00259	0.00143	0.00116
Number of government-issued food safety regulations (X7)	−0.0545 **	0.0130 **	0.0415 **	−0.0741 ***	0.0102 ***	0.0639 ***	−0.0640 ***	0.0352 ***	0.0287 ***
Effectiveness of government-issued food safety regulations (X8)	−0.0796 ***	0.0190 ***	0.0606 ***	−0.0373 *	0.00514 *	0.0322 *	−0.00648	0.00357	0.00291
Number of platform-issued food safety regulations for online catering (X9)	−0.00428	0.00102	0.00326	−0.00391	0.000538	0.00337	−0.0273	0.0150	0.0123
Effectiveness of platform-issued food safety regulations (X10)	−0.0173	0.00413	0.0132	0.00716	−0.000985	−0.00617	−0.0351 *	0.0193 *	0.0157 *
Frequency of restaurant staff training on food safety by restaurant owners (X11)	−0.0470 **	0.0112 **	0.0358 **	0.0242	−0.00333	−0.0209	0.0109	−0.00598	−0.00488
Employee food safety awareness (X12)	0.00976	−0.00233	−0.00743	−0.0388 *	0.00534 *	0.0335 *	−0.0374 **	0.0206 **	0.0168 *
Cleanliness of courier delivery box (X13)	−0.0454 **	0.0108 *	0.0345 **	−0.0959 ***	0.0132 ***	0.0827 ***	−0.0960 ***	0.0529 ***	0.0431 ***
Courier personal hygiene (X14)	−0.0617 ***	0.0147 ***	0.0470 ***	−0.0528 ***	0.00726 **	0.0455 ***	−0.0833 ***	0.0459 ***	0.0374 ***
Trust in platform services (X15)	−0.0408 **	0.00975 *	0.0311 **	−0.0256	0.00352	0.0221	−0.108 ***	0.0598 ***	0.0487 ***
Confidence in government supervision (X16)	−0.0156	0.00372	0.0119	−0.0638 ***	0.00878 **	0.0550 ***	−0.0781 ***	0.0430 ***	0.0351 ***

Notes: t statistics in parentheses. * *p* < 0.10, ** *p* < 0.05, *** *p* < 0.01.

## Data Availability

The original contributions presented in the study are included in the article, further inquiries can be directed to the corresponding authors.
